# Structural and physicochemical properties of rice starch from a variety with high resistant starch and low amylose content

**DOI:** 10.3389/fnut.2024.1413923

**Published:** 2024-05-27

**Authors:** Xue Gu, Peng Wang, Juyuan Huang, Shuangqin Chen, Dandan Li, Shihuang Pu, Juan Li, Jiancheng Wen

**Affiliations:** ^1^Rice Research Institute, Yunnan Agricultural University, Kunming, China; ^2^College of Agronomy and Biotechnology, Yunnan Agricultural University, Kunming, China

**Keywords:** rice varieties, resistant starch, starch structure, physicochemical properties, starch digestion characteristics

## Abstract

Research on the physicochemical properties of rice-derived endo-sperm high resistant starch (RS) with low amylose content (AC) is limited. In this study, we evaluated the physicochemical characteristics of such a starch variety and revealed that the starch granules exhibit a smoother, more refined surface with distinct edges, increased compactness, higher order of surface, and fewer cavities compared to those of a low RS rice variety. The starch crystal was classified as an A-type, which may be connected to the high amylose-lipid complex content. The branched internal long chains (B2 + B3) were abundant, allowing for easy entanglement with other molecular chains and a compact structure. Differential scanning calorimetry revealed the need for high temperature and energy to disrupt the double helix structure within the crystallization region of starch. Furthermore, starch viscosity analysis revealed a high cold paste viscosity, consistency, and setback value, with recrystallization yielding a stable structure, increased viscosity, and enhanced hydrolysis resistance to enzymes.

## 1 Introduction

Starch is classified as rapidly digestible starch (RDS), slowly digestible starch (SDS), or resistant starch (RS), depending on its rate of postprandial digestion. RDS are starch molecules that fully digest into glucose within 20 min owing to the production and quick release of glucose during digestion and absorption. Such a process increases the blood glucose levels of consumers immediately after consuming certain foods high in carbohydrates. Conversely, SDS requires 20–120 min of enzymatic hydrolysis to be digested and absorbed in the small intestine. SDS complete digestion occurs at a significantly slower rate than RDS, which results in a delayed release of glucose from hydrolyzed starch and a gradual rise in blood glucose levels after digestion (1). RS resists digestion and absorption in the small intestine of healthy individuals (2) and is beneficial to intestinal health owing to its fermentative interaction with colon microorganisms that generate short-chain fatty acids (3–5). RS can improve intestinal dysfunction, reduce intestinal disease incidence, lower postprandial blood glucose levels, and prevent and improve many health issues such as diabetes, obesity, and cardiovascular diseases (6–9). Prolonged consumption (over 8 weeks) of RS-rich foods effectively improves blood glucose levels (10). Given the increasing global prevalence of diabetes due to shifts in dietary patterns and lifestyles, blood glucose maintenance has become one of the most significant human health concerns (11). Therefore, enhancing the daily intake of RS-rich foods provides a promising public health strategy.

In recognition of the substantial health benefits of RS, crop variant development with an improved RS content has become a conscious goal. The RS content varies notably across different crops; legumes contain up to 36% RS, whereas cereal crops usually contain <3.0% RS (12, 13). Certain RS-high rice varieties have been identified such as Chikushi-kona 85 and Jiangtangdao1 exhibiting 17.4 and 11.6% RS, respectively (14, 15). In addition, the RS_2_ and RS_3_ contents of the high amylose starch mutant Goami2 were 6.28 and 11.45%, respectively (16). The amylose content of rice endosperm is positively correlated with its RS content (17–19), especially in rice carrying the *Wx^a^* allele which produces high amylose content that is significantly higher than that of rice carrying the *Wx* allele (20). This finding has led to high RS rice variants typically having high amylose contents, thereby producing harder and less palatable rice. However, Diangu 2, a variety discovered via the analysis of many rice endosperms, exhibits a high RS content with low amylose content (13), resulting in softer and tastier rice. Unfortunately, reports on low amylose and high RS rice varieties, and research regarding the physicochemical properties of their starch remain lacking. Thus, this study aimed to analyze the physicochemical characteristics of Diangu 2 starch, which has low amylose and high RS contents, and provide empirical data to aid in the breeding of rice varieties that balance a high RS content with taste.

## 2 Materials and methods

### 2.1 Materials

The indica rice variant Diangu 2, which exhibits a high RS content in its endosperm, and the control variant Diantun 502, which has a low RS content in its endosperm, were provided by the Rice Research Institute of Yunnan Agricultural University. Notably, both variants exhibited low levels of endosperm amylose. The rice was cultivated in Yuanyang County, Yunnan Province. The mature rice was harvested and naturally air-dried, followed by the removal of hollow and imperfect grains. Subsequently, the rice underwent a 15 s milling process using a small rice pearling mill, followed by pulverization into a fine powder and sieving through a 100-mesh sieve. The Total Starch (TS) Assay Kit and the D-Glucose Assay Kit (GOPOD format) were purchased from Megazyme International Ireland Limited, which contains thermostable α-amylase, amyloglucosidase, glucose oxidase/peroxidase reagent (GOPOD) enzyme, and a buffer solution. Pancreatin (P1750, 12,450 U), α-amylase (10,080, 50 U/mg), pepsin (P6887, ≥ 3,200 U/mg), amylose, and amylopectin standards were purchased from Sigma Chemical Company. All other chemicals and solvents used were of analytical grade.

### 2.2 Isolation of rice starch

Rice starch was isolated following the method described by Syahariza and Hasjim (21). An appropriate quantity of polished rice was mixed with distilled water and left to soak for 24 h at room temperature. The supernatant was then removed by centrifugation at 5,000 g for 20 min, followed by the addition of anhydrous ethanol. The mixture was thoroughly mixed, shaken for 10 min, and then filtered through a 200-mesh sieve (washed with anhydrous ethanol). After centrifugation at 5,000 g for 20 min, the supernatant was discarded and the precipitate was washed three times with distilled water. 1.6 g/L of papain aqueous solution was added and the mixture was thoroughly stirred and shaken for 1 h. The suspension was centrifuged at 5,000 g for 20 min and the supernatant was removed. 0.25% NaOH solution was added in the proper quantity, thoroughly mixed, shaken for 10 min, and then filtered through a 200-mesh sieve (washed with distilled water). After 20 min of centrifugation at 5,000 g, the supernatant was discarded, and the precipitate was washed three times with distilled water, The precipitate was transferred to a clean dish and dried in an oven at 40°C. The starch block was pulverized and passed through a 200-mesh sieve to afford the rice starch.

### 2.3 Amylose content

The amylose content of rice was analyzed according to the methods presented in China National Standard “Rice - Determination of amylose content” (22).

### 2.4 Total starch content

The quantification of the TS content was performed using the Megazyme Total Starch Assay Kit (AOAC, 996.11). The rice flour sample (100 mg) was placed in a 15-mL centrifuge tube and 0.2 mL of 80% v/v aqueous ethanol was added together with 2 mL of 1.7 M NaOH solution. The mixture was placed in an ice/water bath and stirred for 15 min with a magnetic stirrer. During this period, the mixture was intermittently vortexed 2–3 times to remove all lumps from the sample slurry. Sodium acetate buffer (8 mL; 600 mM, pH 3.8, and 5 mM CaCl_2_) was added, followed by homogenization. Undiluted thermostable α-amylase (0.1 mL) was added, followed by the addition of AMG (0.1 mL, 3,300 U/mL). The mixture was vortexed for 3 s and then incubated at 50°C for 30 min. Portions of (2.0 mL) of the mixture were transferred to centrifuge tubes and centrifuged at 5,000 g for 5 min. The supernatant (1.0 mL) was pipetted to a 12 mm × 120 mm test tube containing 10 mL of 100 mM sodium acetate buffer (pH 5.0) and was homogenized. Finally, the D-Glucose assay kit was used to determine the glucose content of each sample.

### 2.5 Starch composition

The starch composition was determined following the method reported by Englyst et al. (2). The rice flour sample (50 mg) was accurately weighed into a centrifuge tube and 2 mL of distilled water together with three glass beads were added. The mixture was placed in a boiling water bath for 20 min and was vortexed several times during this period. Artificial saliva α-amylase (1 mL) was added to the mixture and the starch was digested for 2 min. Digestion with 5 mL of a pepsin solution was then performed in a shaking water bath at 37°C and 200 rpm for 30 min. The digestion fluid was neutralized and the pH was adjusted by adding 5 mL of a 0.02 M sodium hydroxide solution and 20 mL of a 0.2 M sodium acetate buffer. The trypsin-AMG mixed enzyme solution (5 mL) was added and digestion was performed for 2 h in a shaking water bath (set to 37°C and 200 rpm). During digestion, a portion (1 mL) of the mixture was sampled at three times points (0, 20, and 120 min); 1 mL of absolute ethanol was added to each sample, followed by centrifugation at 4000 rpm for 5 min. The supernatant was then used for glucose content determination using the D-Glucose Assay Kit. Finally, the RDS, SDS, and RS contents of the endosperm were determined using the following formulas:

RDS%=[(G20−FG)×0.9/TS]×100.

SDS%=[(G120−G20)×0.9/TS]×100.

RS%=[(TS−RDS−SDS)/TS]×100.

where G20 and G120 represent the glucose released within 20 and 120 min, respectively, whereas FG and TS represent the free glucose and total starch, respectively.

### 2.6 Scanning electron microscopy (SEM)

Distilled water (1 mL) was added to 100 mg starch in a 2-mL centrifuge tube. The mixture was vortexed until uniformly mixed, centrifuged at 5,000 g for 1 min, and the supernatant was discarded. This process was repeated five times. Finally, 1 mL of anhydrous ethanol was added to the tube followed by vortexing until evenly mixed. A small amount of the prepared starch suspension was placed onto the conductive adhesive on a copper plate using a 200-μL pipette. The suspension was evenly spread and left overnight at 37°C. After sputtering gold ions with an ion sputtering equipment, the sample was observed under the microscope.

### 2.7 Branched chain length distribution of amylopectin

The chain length distribution of the starch sample was analyzed using high-performance anion-exchange chromatography (HPAEC-PAD) on an electrochemical detector (ISC5000+ pulse amperometric detector). A purified starch sample (5 mg) was resuspended in 5 mL of double-distilled water and heated in a boiling water bath for 60 min with periodic vortex mixing to form a gelatinized sample. To a portion of 2.5 mL, sodium acetate (125 μL), NaN_3_ (5 μL), and isoamylase (5 μL) (I5284, Sigma, St. Louis, MO, United States) were added. The mixture was then placed at 38°C for 24 h. Subsequently, 600 μL of the mixture was transferred to a centrifuge tube and dried at room temperature under nitrogen gas. The dried sample was then dissolved in 600 μL mobile phase, centrifuged at 6000 g for 5 min, and the supernatant was used for loading. The sample was then subjected to high-performance anion-exchange chromatography (HPAEC) (ICS5000+, Thermo Fisher Scientific, United States), equipped with a Dionex™ CarboPac™ PA10 (250*4.0 mm, 10 μm) column, at an injection volume of 20 μL. The mobile phase consisted of phase A (200 mM NaOH) and phase B (200 mM NaOH/200 mM NaAC). The column temperature was maintained at 30°C. Thermo Fisher Chromeleon software 7.2 was used to sum the peak areas of sample DP 6-DP 76 to derive the total area, and each peak area was divided by the total area to compute the proportion of the different chain lengths. The relative area was calculated as Relative Area = (Area / Total Area) × 100%.

### 2.8 X-ray diffraction (XRD)

Crystallographic analysis of the sample was conducted using an X’Pert Pro X-ray diffractometer (PANalytical, Netherlands), with the degree of crystallinity, crystal morphology, and characteristic parameters of the diffraction angle 2θ computed via MDI Jade 5.0 software. Sufficiently dried starch samples were used. Cu-Kα (*λ* = 0.15406 nm) radiation emitted from a copper target was used, operating at a power of 1,600 W (40 kV × 40 mA). The intensity of X-rays was determined by a NaI crystal scintillation counter. The scan was conducted over a range of 5°–60°, with a step size of 0.02° and a scan speed of 6°/min.

### 2.9 Fourier transform infrared spectroscopy (FTIR)

A starch sample (~50 mg) was combined with KBr and compressed into a pellet. FTIR analysis was performed using a Nicolet iZ-10 (UK), equipped with a KBr detector, a KBr beam splitter, and an infrared light source. The analytical conditions were set as follows: number of scans, 32; resolution, 4.00 cm^−1^; scanning range, 400–4,000 cm^−1^; sampling gain, 8.0; mirror speed, 0.4747; aperture, 80.00.

### 2.10 Thermodynamic analysis

The thermodynamic properties of the starch samples were analyzed using through differential scanning calorimetry (DSC, Q2000, TA Instruments, United States). Dried and moisture-equilibrated samples were ground in a mortar, and filtered through a 200-mesh sieve, and 10 mg of each sample was accurately weighed and placed in a sealed alumina crucible. Sterile water (30 μL) was added, and after equilibrating at room temperature for 24 h, the samples were loaded into the calorimeter. The temperature was increased from 30 to 95°C at a rate of 10°C/min, and changes in heat capacity were determined. The collected data were analyzed using the Universal Analysis software. The onset temperature (T onset), peak temperature (T peak), conclusion temperature (T conclusion), and gelatinization enthalpy (ΔH) were calculated to characterize the phase transition process of the samples.

### 2.11 Viscosity properties

The gelatinization and viscosity characteristics of the starch samples were assessed using a rapid visco analyzer (RVA-TecMaster, Perten, Sweden). The detection process was performed according to the AACC 61–02.01 method (23). Rice flour samples of 3 g (MC 12%) were accurately measured and placed in a specialized aluminum sample canister designed for RVA use. The samples were tested after adding distilled water (25 mL) and inserting the mixer paddle. The RVA analysis can monitor the starch viscosity in real-time by detecting changes in the starch paste viscosity using a standardized heating-steady temperature-cooling cycle established by AACC, thus producing a curve. The collected data were processed using the TCW3 (Thermocline for Windows-3) software, and parameters such as peak viscosity (PV), hot paste viscosity (HPV), cool paste viscosity (CPV), breakdown viscosity (BDV), setback viscosity (SBV), and consistency viscosity (CSV) were obtained.

### 2.12 Statistical analysis

All analyses were conducted in triplicate, and the mean values and standard deviations were reported. Analysis of the structural characteristic data was performed in Excel 2018, whereas single factor analysis of variance (ANOVA) was performed using Tukey’s HSD test (*p* < 0.05) on SPSS 26.0 software. All graphs were constructed using Origin 2021 and GraphPad Prism software.

## 3 Results

### 3.1 Starch composition

**Figure 1:**
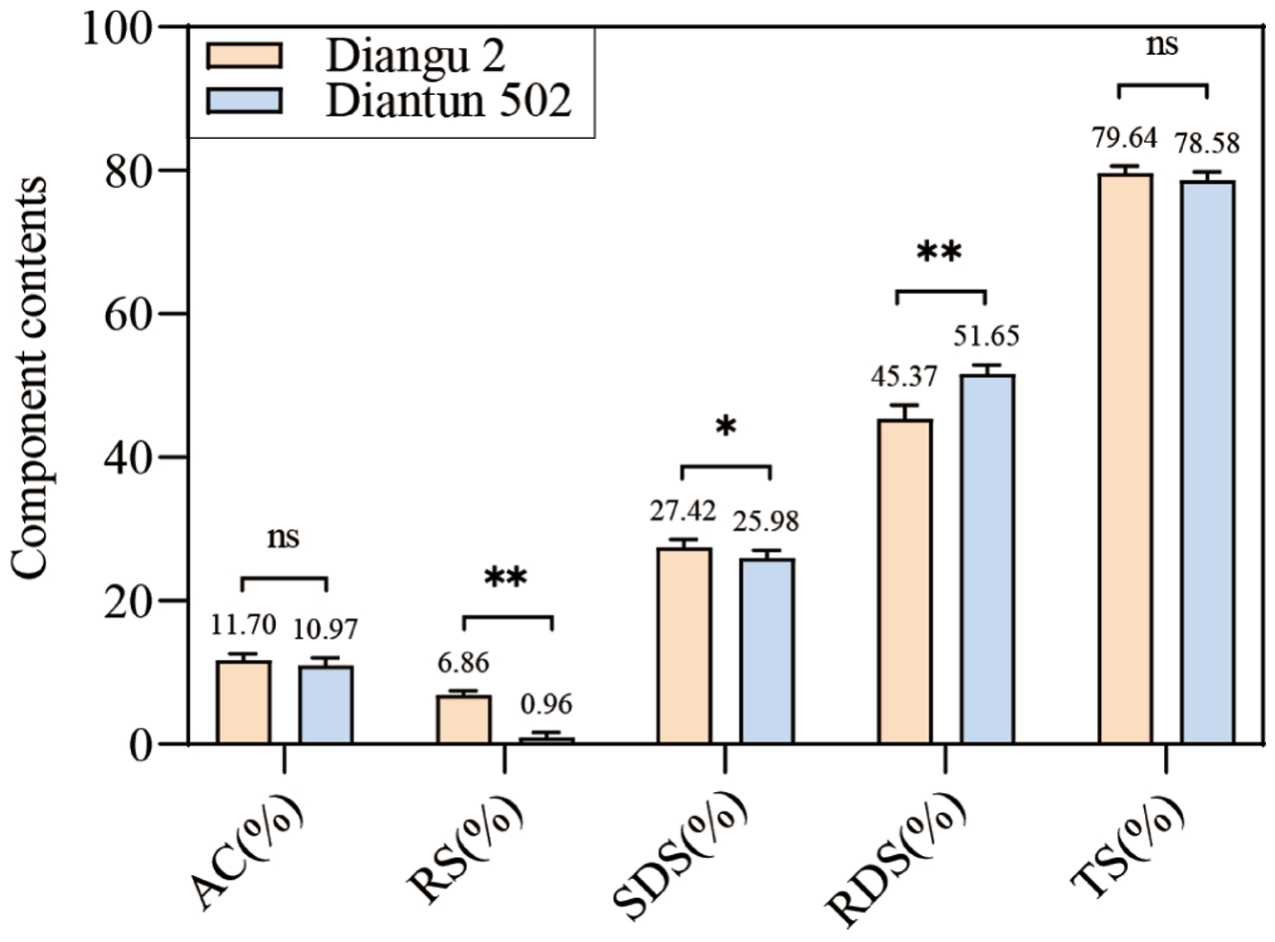
Composition of rice starch. AC, amylose content; RS, resistant starch; SDS, slowly digestible starch; RDS, rapidly digestible starch; TS, total starch. ^**^p < 0.01; ^*^p < 0.05; ns: *p* > 0.05; the error bars represent ± standard deviation (SD) (*n* = 3).

The RS and SDS contents in the endosperm of the Diangu 2 rice variety were 6.86 and 27.42%, respectively, which are significantly higher than those observed in Diantun 502 (0.96 and 25.98%, respectively, *p* < 0.01 and *p* < 0.05, respectively). The RDS content of the Diangu 2 endosperm was 45.37%, significantly lower (*p* < 0.01) than the 51.56% observed in Diantun 502 (Figure 1). In addition, the amylose contents in the endosperms of Diangu 2 and Diantun 502 were 11.7 and 10.97%, respectively, classifying them as low-amylose varieties, thereby yielding soft and palatable rice.

### 3.2 SEM analysis

**Figure 2:**
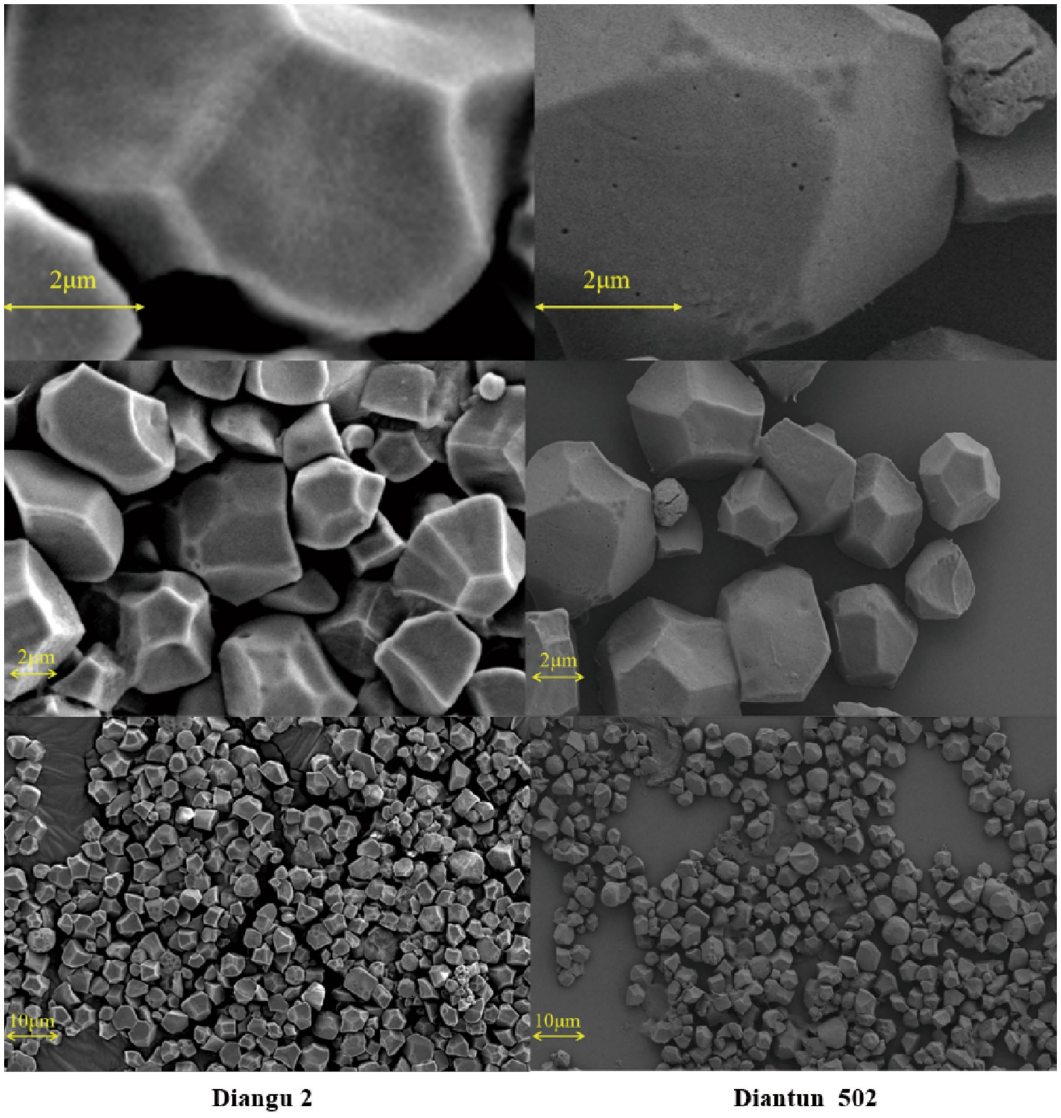
Scanning electron microscopy (SEM) images of starch granules in rice endosperm. Left: starch granules in the endosperm of Diangu 2. Right: starch granules in the endosperm of Diantun 502.

The morphological features and surface characteristics of starch granules extracted from the rice varieties Diangu 2 and Diantun 502 are shown in Figure 2. The starch granules in Diangu 2 predominantly exhibit irregular polyhedral forms, with a few displaying a regular prismatic shape. Conversely, the Diantun 502 starch granules predominantly exhibit polygonal and prismatic shapes, with a few round granules. Consistent with previous research findings (24), the diameters of starch granules from both rice varieties ranged between 2 and 10 μm. However, a significant difference (*p* < 0.05) was observed in the surface characteristics of the granules: the Diangu 2 starch granules presented a refined surface, displaying clear edges, corners, and few pores. In contrast, the Diantun 502 starch granules featured a rough, loose surface with microscopic pores.

### 3.3 Amylopectin branched chain length distribution

**Figure 3:**
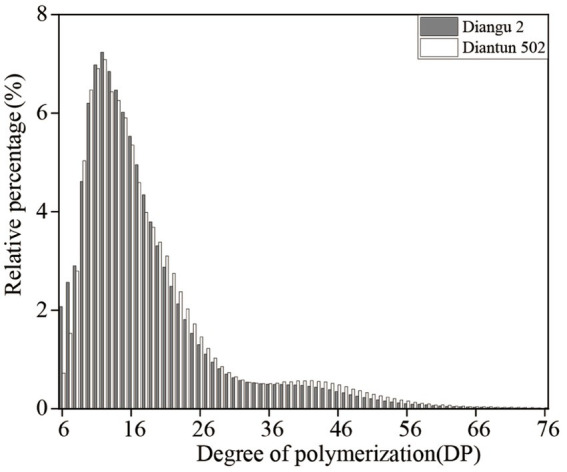
Distribution of the branched chain lengths of amylopectin of starch.

The branched chain length distribution of the starch derived from the rice varieties Diangu 2 and Diantun 502 exhibited comparable distribution curves, each manifesting similar bimodal characteristics. The first peak is observed at approximately DP12, while the second peak occurs around DP42. As the degree of polymerization (DP) of the amylopectin chain length escalates, its relative proportion initially increases and then decreases (Figure 3). Despite the similarities, the peak values between the two varieties vary, and a discrepancy is observed in the proportion of the DP of amylopectin (Table 1). The relative proportions of short A chains (DP6-12) and short chains (A + B1) in Diangu 2 are 30.54 and 80.37%, respectively, which are significantly lower (*p* < 0.05) than the corresponding proportions of 32.62 and 83.10% in Diantun 502. Conversely, the proportion of long chains (B2 + B3) in Diangu 2 is 19.22%, which is significantly higher (*p* < 0.01) than the 16.89% observed in Diantun 502. No significant difference was observed in the short B1 chains, long B2 chains, long B3 chains, and the average amylopectin DP between the two rice varieties.

**Table 1:** Distribution of the branched chain length of amylopectin of starch.

Degree of polymerization	Diangu 2	Diantun 502	*F* value
DP6-12 (A)	30.54 ± 1.19	32.62 ± 0.45	7.968^*^
DP13-24 (B1)	49.83 ± 0.22	50.47 ± 0.36	7.059 ns
DP25-36 (B2)	10.50 ± 0.22	9.53 ± 0.86	3.621 ns
DP > 36 (B3)	8.72 ± 0.76	7.36 ± 0.43	7.255 ns
Short chain (A + B1)	80.37 ± 1.41	83.10 ± 0.80	8.454^*^
Long chain (B2 + B3)	19.22 ± 0.65	16.89 ± 1.28	7.892^**^
Average degree of polymerization	18.81 ± 0.21	18.04 ± 0.18	4.784 ns

^*^p < 0.05; ns, not significant *p* > 0.05; mean ± standard deviation (SD) represents a repetition of three experiments.

### 3.4 XRD analysis

**Figure 4:**
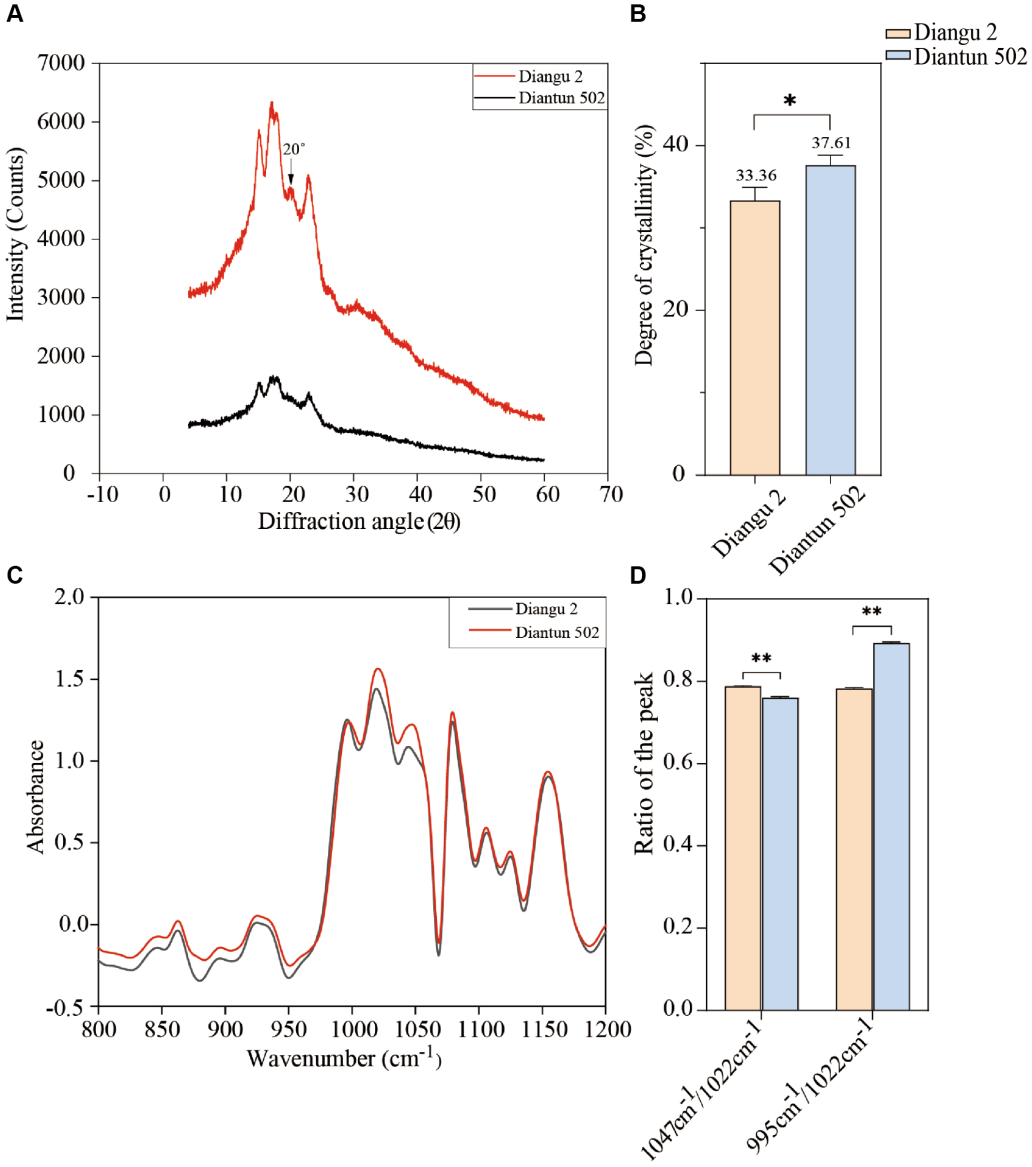
Structural properties of starch from rice varieties. **(A)** X-ray diffraction (XRD) spectra. **(B)** Difference of crystallinity. **(C)** Fourier transform infrared spectroscopy (FTIR). **(D)** Difference of infrared spectrum resonance peak ratio. ^**^p < 0.01; ^*^p < 0.05; ns: *p* > 0.05; the error bars represent ± standard deviation (SD) (*n* = 3).

The XRD spectra, spanning a diffraction angle (2θ) of 4°–60°, display comparable spectral attributes exhibited prominent absorption peaks at 15°, 17°, 18°, and 23° for both starch varieties (Figure 4A). The Diangu 2 starch exhibited another pronounced absorption peak of approximately 20° compared to that observed in Diantun 502. Compared to the Diantun 502 starch, the diffraction peaks of the Diangu 2 starch are notably taller and sharper, suggesting the presence of larger microcrystalline particles and a more compact structure. As shown in Figure 4B, the relative crystallinity of Diangu 2, a high RS variety, was 33.36%, which is significantly lower (*p* < 0.05) than the 37.61% observed in Diantun 502, a low RS variety.

### 3.5 FTIR analysis

As shown in the FTIR spectra (Figure 4C), the characteristic absorption peaks at 1047 cm^−1^ (or 1,045 cm^−1^) and 1,022 cm^−1^ are higher in Diangu 2 than in Diantun 502, while the absorption peak at 995 cm^−1^ does not differ notably between the two varieties. When considering the overall peak shape, both varieties present similar infrared absorption spectra, and the positions of their absorption peaks align, suggesting a similar chemical configuration in these two rice starch granules without discernable group differences. The peak ratio at 1047 cm^−1^ (or 1,045 cm^−1^)/1,022 cm^−1^ for the Diangu 2 starch is significantly higher (*p* < 0.01) than that of Diantun 502. Conversely, its peak ratio at 995 cm^−1^/1022 cm^−1^ is significantly lower (*p* < 0.01) than that of Diantun 502 (Figure 4D).

### 3.6 Thermodynamic analysis

**Figure 5:**
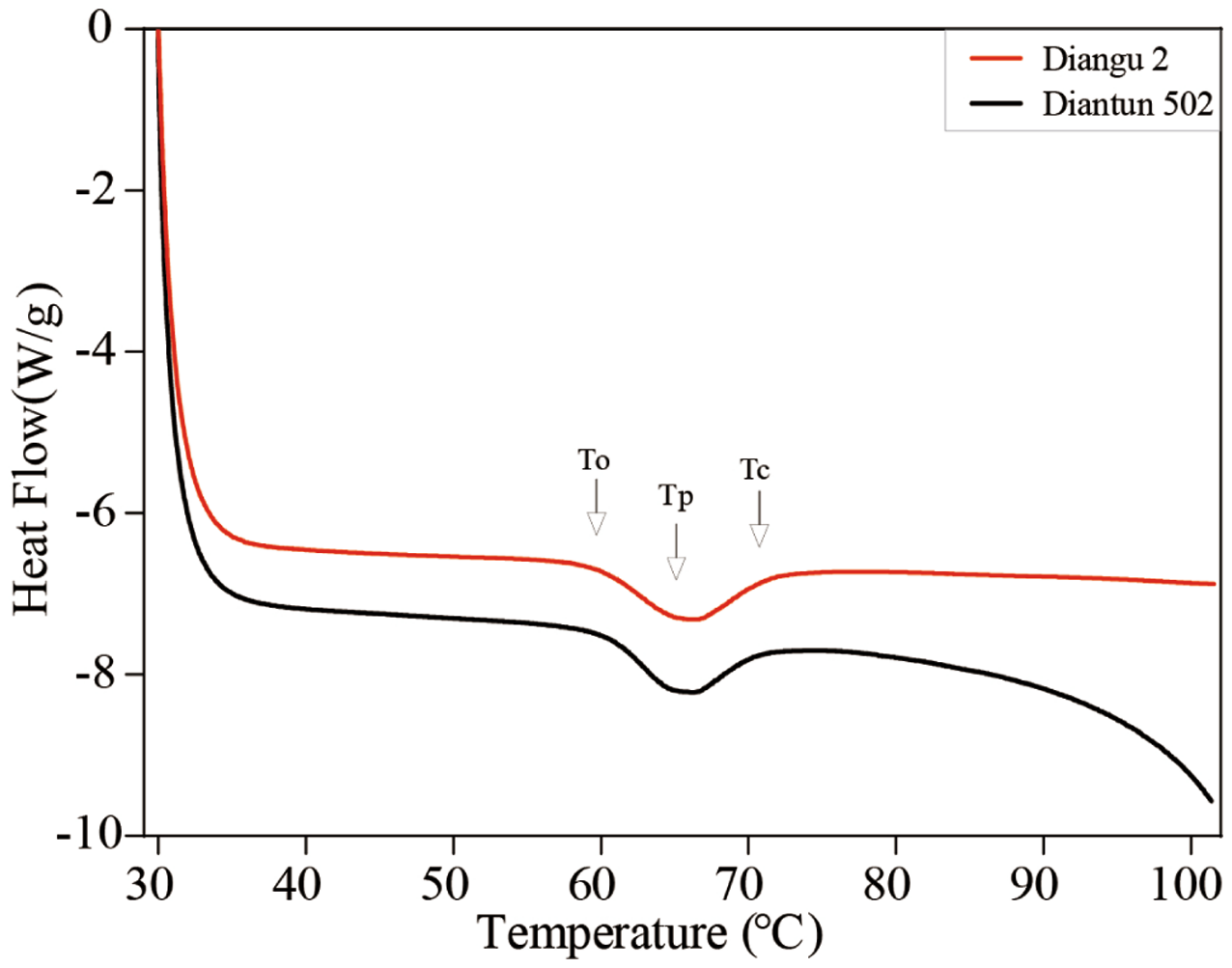
Differential scanning calorimetry (DSC) thermodynamic property curve of starch.

According to the DSC thermodynamic property curves, the two types of starch granules absorb heat and start to paste when the double helix on the outer side chain of the branching crystal structure dissociates at approximately 60°C (25), which marks the onset temperature (Figure 5). An absorption peak appears at ~65°C, indicating the peak temperature. After this peak, the rate of heat absorption decelerates and the gelatinization is completed at ~71°C, where the DSC curve flattens once again. As shown in Table 2, the peak temperature, gelatinization temperature range, and gelatinization enthalpy of the Diangu 2 starch significantly exceeded those of Diantun 502. This suggests a more uniform and intact crystal structure in the Diangu 2 starch (compared to Diantun 502), requiring higher temperatures and energy levels to disrupt the double helix structure within its crystalline region.

**Table 2:** Comparison of differential scanning calorimetry (DSC) thermodynamic properties of starch.

Parameter	Diangu 2	Diantun 502	*F* value
Onset temperature To (°C)	59.96 ± 0.97	60.44 ± 0.68	0.497 ns
Peak temperature Tp (°C)	65.89 ± 0.57	64.65 ± 0.52	7.767^*^
Crystallization temperature Tc (°C)	71.09 ± 0.41	70.49 ± 0.77	1.455 ns
Range of gelatinization temperature ΔT (°C)	11.13 ± 0.59	10.04 ± 0.14	9.465^*^
Gelatinization enthalpy ΔH (J/g)	11.72 ± 0.70	9.41 ± 0.51	21.218^**^

^*^p < 0.05; ^**^p < 0.01; ns: not significant p > 0.05; mean ± standard deviation (SD) represents a repetition of three experiments.

### 3.7 Viscosity analysis

**Figure 6:**
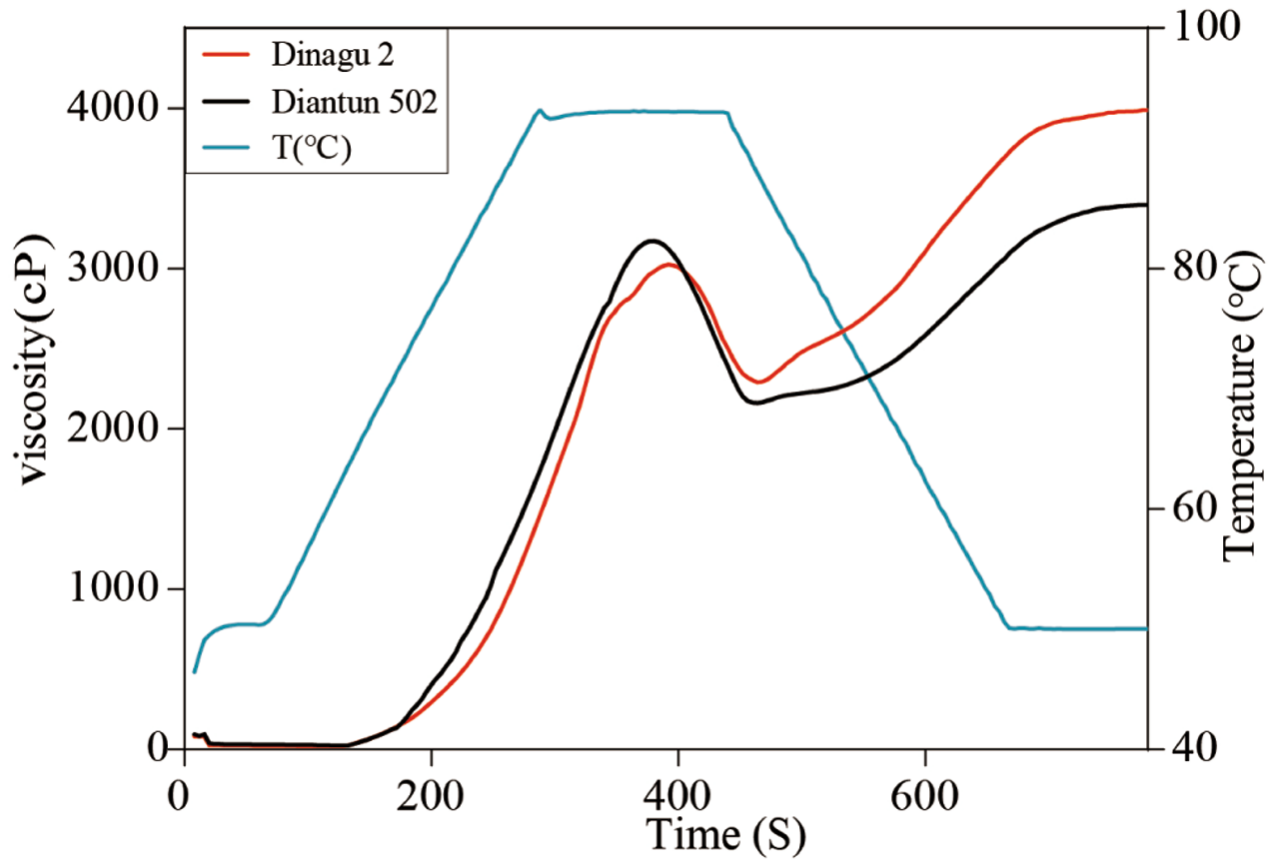
Rapid visco analyzer (RVA) curves indicating differences of starch.

The RVA curves of starch derived from Diangu 2 and Diantun 502 follow the same trend (Figure 6). The gelatinization temperature, peak time, retrogradation, and recrystallization time of the starch granules show small differences between the two variants. However, significant differences are evident between the peak viscosities of the RVA curves for the starch granules of the two varieties. The HPV of the Diangu 2 starch granules was significantly higher (*p* > 0.05) than that of Diantun 502 (Table 3). The CPV, consistency viscosity, and setback viscosity of Diangu 2 were significantly higher (*p* > 0.01) than those of Diantun 502. Conversely, its breakdown viscosity was significantly lower (*p* < 0.01) than that of Diantun 502. Moreover, compared to Diantun 502, Diangu 2 exhibited lower PV, peak time, and gelatinization temperature.

**Table 3:** Differences in rapid visco analyzer (RVA) characteristic values of starch.

Characteristic value	Diangu 2	Diantun 502	*F* value
Peak viscosity (PV)	3039.67 ± 67.21	3141.00 ± 27.81	5.961 ns
Hot paste viscosity (HPV)	2194.67 ± 62.63	1992.33 ± 60.87	16.101^*^
Cool paste viscosity (CPV)	3968.00 ± 41.33	3333.33 ± 57.57	240.606^**^
Breakdown viscosity (BDV)	845.00 ± 4.58	1148.67 ± 33.86	236.985^**^
Consistency viscosity (CSV)	1773.33 ± 52.54	1341.00 ± 35.01	140.629^**^
Peak time (PeT)	385.33 ± 8.32	376.00 ± 4.00	3.062 ns
Setback viscosity (SBV)	928.33 ± 56.08	192.33 ± 40.28	340.856^**^
Pasting temperature (PaT)	69.73 ± 0.03	70.02 ± 0.47	1.074 ns

^*^p < 0.05; ^**^p < 0.01; ns: not significant *p* > 0.05; mean ± standard deviation (SD) represents a repetition of three experiments.

## 4 Discussion

### 4.1 Relationship between starch structure and resistance to enzymatic degradation

A significant positive correlation between the RS and amylose contents has been previously established (17–19), and some studies have reported that high-RS rice varieties are also high-amylose varieties (26). However, the amylose contents in the endosperms of Diangu 2 and Diantun 502 were low-amylose varieties, thereby yielding soft and palatable rice. This soft rice with a more palatable taste, contrasts the findings of the previous study.

RS, SDS, and RDS affect the rate of starch hydrolysis and glucose release, having an impact on the glycemic index (27). RS inhibits the release of glucose, which minimizes the blood glucose response and helps to prevent high blood glucose levels. Kwak et al. (28) used rice containing resistant starch to dietary treatment patients with impaired fasting blood glucose, impaired glucose tolerance, and newly diagnosed type II diabetes, reducing their fasting insulin, postprandial blood glucose, and insulin levels. SDS helps to maintain stable blood glucose levels, therefore reducing feelings of hunger. In a test of an SDS-enriched diet, Gou et al. (29) found that insulin-resistant subjects showed lower postprandial glucose and insulin responses and that SDS had a significant effect on improving insulin sensitivity. During processing, increasing the SDS and RS content of the product reduces starch digestibility and can improve the nutritional quality of the grain (30). Hence, consuming foods rich in RS and SDS can reduce the risk for many chronic diseases (31). In contrast, RDS is quickly absorbed in the small intestine, and a high RDS content will rapidly increase postprandial blood glucose; therefore, it is unsuitable for people with diabetes (32, 33). Consuming Diangu 2 is healthier than Diantun 502 because it contains more RS, SDS, and less fast digestible starch.

The morphological features of starch granules profoundly influence the natural raw starch digestion and absorption. The pores and channels on the granule can affect amylase adsorption and binding (34). Various studies have reported that cereal starch, characterized by its rough surface and microscopic pores, is readily digested, whereas potato starch, due to its smooth surface and lack of pores, is digestion-resistant (35). Interestingly, Diangu 2, a cereal crop, exhibited starch granule surface features akin to those of potato starch. The smooth surface of the starch granules of Diangu 2 hampers amylase adsorption, thereby reducing its adsorption rate. Moreover, it is difficult for amylase to enter the interior of these compact granules, and hydrolysis can only be initiated from the granule surface, reducing the hydrolysis rate. These factors contribute to the enhanced resistance of Diangu 2 starch to enzymatic hydrolysis, rendering its digestion difficult.

The structural properties of starch are largely influenced by the distribution of the amylopectin chain length. This influence is primarily observed in the interactions among glucans of different branches within amylopectin and between the chains of amylopectin and amylose molecules. Previous research revealed that the short-chain constituents in amylopectin are not involved in starch crystallization (36), however, a short-chain abundance disrupts the anti-enzymatic digestion of microcrystals. Conversely, the amylopectin molecules are characterized by a scarcity of short chains and a large number of long chains are predisposed to form relatively intact anti-digestive microcrystals (37). According to previous reports, the RS3 level increases with the length of the starch chain (38). Higher RS levels and delayed hydrolysis were observed in the intestinal digestion stage of the indica glutinous rice Zongzi, which had a longer chain level (39). In this study, the starch of the Diangu 2 variety exhibited a smaller proportion of short A and B1 chains and a larger proportion of long B2 and B3 chains. Such a molecular chain ratio is prone to entanglement, which enhances the rotation radius of the molecular chain and the double helix structure. This leads to anti-digestive microcrystal structure formation, resulting in a high RS content.

The X-ray diffraction spectra of starch granules reflect the crystal structure and crystallinity of starch, Prominent absorption peaks at 15°, 17°, 18°, and 23° suggest that the starch crystal structure and crystallinity align with the typical features of Type A crystals for both varieties (40). The Diangu 2 starch exhibited a pronounced absorption peak of approximately 20° compared to that observed in Diantun 502, implying the potential abundance of amylose-lipid complexes. This absorption peak has been previously associated with amylose-lipid complexes (41), and their existence is closely related to the resistance of starch to enzymatic hydrolysis (42, 43). In XRD spectra, a diffraction peak that is tall and narrow indicates large microcrystalline particles and a dense structure. In contrast, a broad and short diffraction peak signifies small microcrystalline particles and a loose structure (44). Compared to Diantun 502, Diangu 2 had a higher RS content, however, it exhibited less relative crystallinity. This finding is consistent with previous reports suggesting that the crystallinity of starch does not necessarily correlate with its RS content and enzymatic resistance (30, 45). The enzymatic resistance and stability of the Diangu 2 starch are not solely derived from the dense structure of the crystalline region. However, these factors may be attributable to its amorphous components that are enveloped in imperfect microcrystals, providing enzymatic resistance, or due to the presence of double helix structures within the amorphous regions of the starch granules (46).

While FTIR spectroscopy cannot discriminate between crystalline forms of starch granules, it is sensitive to the short-range ordering of the particles, such as chain conformation, double helix structure, and helicity (particularly in the 900–1,300 cm^−1^ range) (45). The absorption peak at 1047 cm^−1^ (or 1,045 cm^−1^) is associated with the ordered structure observed within the crystalline region of starch. The absorption peak at 995 cm^−1^ is characteristic of the double helical structure of carbohydrates, signifying hydrated crystals (47). Furthermore, the peak at 1022 cm^−1^ corresponds to the characteristic absorption of the amorphous region in starch granules (48). Although the internal double helical structures of Diangu 2 and Diantun 502 exhibit minimal differences, their starch granules exhibit differences in their crystalline and amorphous regions. In addition, a higher peak ratio (1,047 cm^−1^ or 1,045–1,022 cm^−1^) implies a greater order degree on the surface area of the starch granule, while a higher (995–1,022 cm^−1^) peak ratio suggests a higher double helix prevalence within the starch molecule (49). Thus, based on the peak ratio, it can be inferred that the surface area of the Diangu 2 starch granule exhibits a high order and compact structure. However, the number of double helix structures in its internal amorphous region is smaller than that in Diantun 502.

### 4.2 Impact of starch structure on its physicochemical characteristics

Starch gelatinization is a process where water molecules disrupt the chemical bonds interlinking the starch granules when they are co-heated with water. This results in hydrogen bond cleavage between the molecular chains of starch within both the amorphous and crystalline regions, subsequently leading to the formation of hydrophilic colloidal solutions (50). Differential calorimetric scanning (DSC) was used to determine the pasting characteristics of starch. Among the DSC thermodynamic properties, the peak temperature serves as an indicator of the optimal temperature at which the starch granule structure undergoes maximal heat absorption during the destruction of its crystal structure. A higher peak temperature signifies a greater temperature requirement for crystal structure disruption (51). The gelatinization enthalpy provides a measure of the energy absorbed by the starch granules throughout the gelatinization process, reflecting the energy requirement for starch granule destruction (52, 53). The Diangu 2 starch exhibited a higher peak temperature, gelatinization temperature range, and gelatinization enthalpy compared to the Diantun 502 starch. Such findings indicate that the Diangu 2 starch has a more compact structure (compared to Diantun 502), requiring higher temperatures and energy levels to disrupt it. These characteristics are attributable to the interaction between amylose and the side chains of amylopectin that facilitate efficient packing of the double helix structure. This allows for a more compact crystal structure and enhances the resistance of starch granules to enzymatic digestion (54).

RVA analysis can reveal the starch viscosity properties, which are directly linked to its structure. The structure is mainly affected by the amylose content and amylopectin chain length distribution (55). PV generally indicates the energy and degree to which starch granules bind with water molecules (56). CPV is associated with the ability of starch to form a gel after cooking and cooling, demonstrating a positive correlation with the extent of long amylopectin chain entanglement, and so on (57). The breakdown viscosity reflects the starch fragility, indicating their disruption difficulty. The setback viscosity and consistence viscosity reflect the retrogradation trend and the dehydration condensation capability of the starch paste following complete gelatinization, respectively (58). In summary, the starch granules of Diangu 2 (a variety noted for its high RS content) are larger, more robust, and exhibit greater thermal stability than those of Diantun 502. The Diangu 2 starch molecules resist fracturing during the cooking process, and an extended exposure period is required to attain PV. This attribute is potentially associated with the presence of amylose-lipid complexes within the Diangu 2 starch (59). Furthermore, Diangu 2 shows elevated consistency and setback values, suggesting that during the retrogradation process of the Diangu 2 starch paste, a high degree of reaggregation is observed among the molecular chains of amylose and amylopectin long chains or the long chains of different amylopectin molecules. This recrystallization process yields a stable structure with remarkable viscosity (60, 61).

## 5 Conclusion

Compared to high-RS rice varieties that have a large amylose content, Diangu 2, a high RS rice variety with low amylose content, offers superior taste quality and a softer texture. Furthermore, Diangu 2 presents numerous significant physiological functions that are advantageous to human health.

In this study, we revealed that the starch granules of Diangu 2 exhibited a smoother, more refined, and highly ordered surface with distinct edges, increased compactness, and fewer cavities. The branched internal long chains (B2 + B3) were abundant, allowing for easy entanglement with other molecular chains and a compact structure. The X-ray diffraction pattern identified an A-type starch crystal, with a strong absorption peak near 20°, potentially indicating a high amylose-lipid complex content. DSC revealed elevated peak temperature, gelatinization temperature range, and gelatinization enthalpy, indicating the requirements for a high temperature and energy to disrupt the double helix structure within the crystallization region. Furthermore, starch viscosity analysis demonstrated high CPV, consistency, and setback viscosity, with recrystallization yielding a stable structure.

Facile starch hydrolysis and digestion relies on the morphological attributes of the starch granules surface and its corresponding internal structure. Enzymatic hydrolysis resistance and stability derive from the compact configuration of the crystalline regions of starch. The combination of smooth surfaces and tight internal structures in the Diangu 2 starch granules collectively impede the adsorption and hydrolytic activity of amylase. This consequently amplifies the capacity of Diangu 2 to withstand amylase digestion, reducing absorption and utilization by the human body. As such, while prioritizing human health, there is no need to compromise the delightful taste of rice, offering a superior alternative for rice consumers. Our study findings offer valuable insights for rice variety selection with a high resistant starch content and improved taste.

## Data availability statement

The original contributions presented in the study are included in the article/Supplementary material, further inquiries can be directed to the corresponding authors.

## Author contributions

XG: Conceptualization, Data curation, Formal analysis, Writing – original draft. PW: Investigation, Software, Writing – original draft. JH: Investigation, Validation, Writing – original draft. SC: Investigation, Visualization, Writing – original draft. DL: Funding acquisition, Validation, Writing – original draft. SP: Investigation, Resources, Writing – original draft. JL: Conceptualization, Resources, Writing – original draft. JW: Conceptualization, Data curation, Writing – review & editing.
